# Enhanced expression of Vastatin inhibits angiogenesis and prolongs survival in murine orthotopic glioblastoma model

**DOI:** 10.1186/s12885-017-3125-8

**Published:** 2017-02-13

**Authors:** Yi Li, Jun Li, Yat Ming Woo, Zan Shen, Hong Yao, Yijun Cai, Marie Chia-mi Lin, Wai Sang Poon

**Affiliations:** 1 Brain Tumor Centre, Department of Surgery, The Chinese University of Hong Kong, Hong Kong, China; 2 Department of Neurosurgery, Kwong Wah Hospital, Hong Kong, China; 3 Department of Oncology, Affiliated 6th People’s Hospital, Shanghai Jiaotong University, Shanghai, China; 4 Jiangsu Eng. Lab of Cancer Biotherapy, Xuzhou Medical College, Xuzhou, China

**Keywords:** Vastatin, Glioblastoma, Antiangiogenesis, Gene therapy, Chemoresistance

## Abstract

**Background:**

Antiangiogenic therapies are considered promising for the treatment of glioblastoma (GB). The non-collagenous C-terminal globular NC1 domain of type VIII collagen a1 chain, Vastatin, is an endogenous antiangiogenic polypeptide. Sustained enhanced expression of Vastatin was shown to inhibit tumour growth and metastasis in murine hepatocellular carcinoma models. In this study, we further explored the efficacy of Vastatin in the treatment of GB xenografts.

**Method:**

Treatment of Vastatin was carried out using a nanopolymer gene vector PEI600-CyD-Folate (H1). Antiangiogenic effect of Vastatin was tested in vitro by using co-culture system and conditioned medium. An orthotopic GB murine model was established to examine the in vivo therapeutic effect of Vastatin alone treatment and its combination with temozolomide.

**Results:**

Vastatin gene transfection mediated by H1 could target tumour cells specifically and suppress the proliferation of microvessel endothelial cells (MECs) through a paracrine inhibition manner. Enhancing Vastatin expression by intracerebral injection of H1-Vastatin significantly prolonged animal survival from 48 to 75 days in GB murine model, which was comparable to the effect of Endostatin, the most studied endogenous antiangiogenic polypeptide. The diminished presence of CD34 positive cells in the GB xenografts suggested that Vastatin induced significant antiangiogenesis. Moreover, a synergistic effect in extending survival was detected when H1-Vastatin was administered with temozolomide (TMZ) in GB chemoresistant murine models.

**Conclusion:**

Our results suggest, for the first time, that Vastatin is an antiangiogenic polypeptide with significant potential therapeutic benefit for GB. H1-Vastatin gene therapy may have important implications in re-sensitizing recurrent GB to standard chemotherapeutic agents.

## Background

Glioblastoma (GB) is a lethal and aggressive human malignancy, accounting for over 60% of high-grade primary brain tumours [1, 2]. In spite of significant technological advances in neurosurgery, anaesthesia, intensive care and oncology in the last few decades, GB remains incurable with a median overall survival of 15 months after its first diagnosis [3, 4]. Antiangiogenesis is a therapeutic strategy aiming at the suspension of tumour cells in a state of dormancy by disrupting their blood supply [5]. As hypervascularity, characterized by endothelial proliferation, is a hallmark of GB, antiangiogenic therapies are naturally considered potential oncologic treatment options [6]. Studies focused on this therapeutic strategy have led to the development and approval of bevacizumab, a recombinant humanized monoclonal antibody against vascular endothelial growth factor (VEGF), for recurrent GB [7]. However, such clinical trials have produced inconsistent results and the overall benefits of bevacizumab on GB patients are being challenged [8–10]. Moreover, bevacizumab was not recommended for newly diagnosed GB due to its limited survival benefit and common adverse events [11, 12]. Thus there is an urgent need to develop novel alternative antiangiogenic agents with more convincing therapeutic effects.

Vastatin is the C-terminal non-triple-helical (NC1) domain of the type VIII collagen α1 chain. It is an endogenous polypeptide that initially discovered to inhibit the proliferation and migration of bovine aortic endothelial cells [13]. Our recent study proved that Vastatin, which is normally expressed in normal liver tissue, was distinctly absent in hepatocellular carcinoma (HCC) and possessed antiangiogenic properties. Through interfering with proliferation and metabolism of endothelial cells, Vastatin inhibited tumour growth and prevented metastasis in HCC-bearing rats [14]. Concurrently a recombinant form of Vastatin, rhEDI-8 t, was discovered to be an angiogenesis inhibitor with potential therapeutic benefits for retinopathy-related neovascularization [15]. Since collagen VIII expression is known to be increased in brain tumours and participates in angiogenesis, we are interested in determining whether Vastatin could be used for the treatment of other hypervascular malignancies such as GB [16].

An ideal cancer therapeutic agent should be able to maintain predominantly high concentrations in the tumour thereby minimizing systemic adverse effects. We previously developed a polyplex-forming plasmid delivery agent, Folate-PEI600-CyD (H1). H1 formed nanoparticles with plasmid DNA and showed high affinity to cancer cells through binding to the folate receptors that enriched on cancer cell surface. It had high transfection efficiency especially on GB cells like U87 and U138 [17–19]. More importantly, H1 demonstrated low cytotoxicity and had little effect on normal cells. In the present study we aimed to test the feasibility of using H1 delivered Vastatin gene for treatment of GB xenografts. We report for the first time that enhancing Vastatin expression by H1 mediated gene transfection induced antiangiogenesis and prolonged survival of GB bearing mice, suggesting a promising treatment candidate for future GB drug development.

## Methods

### Cell lines and Cell culture

The murine tumour-derived microvessel endothelial cells (MECs) SVEC4-10EE2 and human GB cell lines U87MG were purchased from American Type Culture Collection (ATCC). They were maintained in either Minimum Essential Medium (MEM; Gibco) or Dulbecco’s modified Eagle’s medium (DMEM; Gibco) with 10% fetal-bovine-serum (FBS; Gibco) supplementation at 37 °C, 5% CO_2_, and used for test within 20 passages after purchase.

GB cells with acquired TMZ resistance (ATR) were derived from U87MG cells through chronic exposure to TMZ. U87MG cells were first incubated in DMEM containing 20 μM TMZ for 2 weeks, then subcultured into DMEM with 200 μM TMZ. Cells that managed to survive and proliferate in this medium for more than five passages were then collected. The final generated cells were considered resistant to TMZ treatment and named U87-ATR.

### Preparation of H1/DNA Polyplexes

Plasmid pORF-EGFP, pORF-Endostatin and pORF-Vastatin were constructed by inserting DNA fragments encoding EGFP, Endostatin and Vastatin into the multiple cloning sites of the pORF-mcs expression vector (InvivoGen). The secretion of Vastatin and Endostatin protein were mediated by the Igk leader. The encoded gene was further confirmed by DNA sequencing.

The PEI600-CyD-Folate (H1) gene vector was synthesized as previously reported [18]. H1 polymer solution was added to pDNA solution in equal volumes to form the polyplexes. The ratio between the amount of nitrogen in PEI and the amount of phosphate in DNA (N/P ratio) was predetermined at 20. The polyplex suspension was allowed to incubate at room temperature for 15 min before being used for transfection or injection.

### Orthotopic GB Murine Model

Animal studies were performed in accordance with the protocol approved by the Animal Experimentation Ethics Committee of the Chinese University of Hong Kong (CUHK). Female nude mice, 6 to 8 weeks old, were purchased from the laboratory animal services center in CUHK. To establish the murine orthotopic GB model, animals were anaesthetised with ketamine:xylazine (100 mg/kg:10 mg/kg.body weight) and mounted into a stereotaxic frame (Stoelting Co.). A burr hole located 0.5 mm anterior to the coronal suture and 1.2 mm right to the sagittal suture was created. U87MG GB cells or U87-ATR cells were harvested and resuspended in phosphate buffered saline (PBS) to a concentration of 1 × 10^5^ cells/μL. The needle of a Hamilton microsyringe was inserted through the burr hole to a depth of 2.5 mm where the right striatum is located. A total of 2 × 10^5^ cells were slowly injected into this area at a rate of 0.2 μL/min. The needle was slowly withdrawn 5 min after cell injection. The mice were then kept within far infrared lighting cabinets until recovery.

### Gene Expression Test

Total 15 mice bearing U87MG xenografts were used for detection of gene expression after H1-Vastatin treatment. Treatments were performed by intracerebral injecting the H1-Vastatin polyplexes to the same location of tumour cell inoculation and ventricle nearby. A 20 μL volume of H1-Vastatin solution was injected into each mouse at a rate of 0.5 μL/min. This process was performed twice, on day 7 and day 14 post cell-inoculation, to achieve a total dosage of 20 μg plasmid DNA. Mice were sacrificed on day 7 (1 h after the first treatment), 10, 14 (1 h after the second treatment), 17 and 21, with 3 mice each time. The right hemispheres were isolated immediately for measurement of Vastatin mRNA level. Total RNA was extracted from brain tissues using TRIzol® Reagent (Invitrogen) and then reverse transcribed to cDNA with SuperScript® II Reverse Transcriptases (Invitrogen). The cDNA was then subjected to PCR assay and gel electrophoresis. The following primer sequences were used: Vastatin (forward:5’- AAC TAC AAC CCG CAG ACA GG -3’; reverse:5’- TGA ATA GAG CAA CCC ACA CG -3’); Collagen VIII α1 (forward: 5’- ACT CTG TCA GAC TCA TTC AGG C -3’; reverse: 5’- CAA AGG CAT GTG AGG GAC TTG -3’); and GAPDH (forward:5’- GAA TCT ACT GGC GTC TTC ACC -3’; reverse:5’-GTC ATG AGC CCT TCC ACG ATG C -3’).

### Animal survival tests

Total 28 mice bearing U87MG xenografts were used to study the survival benefit of H1-Vastatin single treatment. On day 7 after model establishment, the mice were randomized into four groups, 7 mice for each group, and treated with H1-Vastatin, H1-Endostatin, H1-EGFP or PBS respectively. Treatments were performed using the same protocol for H1-Vastatin in gene expression test. The behaviors and survival of these mice were monitored daily. Mouse was sacrificed and recorded as dead when it lost over 20% of its body weight or exhibited serious behavioral disorders like seizures and limb weakness. The animal survivals after model establishment will be summarized in Kaplan-Meier survival curves.

To test the sensitivities of different model to TMZ treatment, 10 mice bearing U87MG xenografts and 10 mice bearing U87-ATR xenografts were used. On day 7 after model establishment, 5 mice with U87MG xenografts and 5 mice with U87-ATR xenografts were scheduled to be treated with TMZ, while the other 10 mice treated with PBS. TMZ was administered via intraperitoneal (i.p.) injection at a dose of 50 mg/kg/day. TMZ powder was first dissolved in dimethyl sulfoxide (DMSO; Sigma) and diluted with PBS before injection. This treatment was performed five times per week and lasted for 2 weeks. The behaviors and survival of animals were monitored daily as mentioned above.

To examine the combination effect of H1-Vastatin and TMZ, 20 mice bearing U87-ATR xenografts were used. On day 7 after model establishment, the mice were randomized into four groups, 5 mice for each group, and treated with H1-EGFP + PBS, H1-EGFP + TMZ, H1-Vastatin + PBS, or H1-Vastatin + TMZ respectively. Treatment of H1-DNA and TMZ were performed using the same protocols mentioned above. The first TMZ administration was carried out 1 h after the first H1-DNA treatment on day 7. The behaviors and survival of animal were monitored and recorded daily.

### Histology study

Nine mice bearing U87MG xenografts were used for histological study and microvessel density (MVD) analysis. On day 7 after model establishment, animals were divided into three groups, three mice in each group, and received the treatment of H1-Vastatin, H1-EGFP or PBS. All these mice were sacrificed on day 42. Whole brain tissues were collected and processed through 10% formalin fixation and paraffin embedding. The tissue blocks were then cut at 5 μm thickness with a microtome for histological analysis. Tumour structure assessment was performed using Hematoxylin & Eosin (H&E) staining. Angiogenesis in tumour tissues was detected by immunohistochemical staining using rabbit anti-CD34 primary antibody (Abcam) and HRP-linked anti-rabbit secondary antibody (Cell Signaling Technology), in accordance with a previous publication [20]. MVD was calculated by counting the percentage of CD34 positive cells in five randomly chosen high-power fields from each tumour.

### Cell proliferation assay

For proliferation assays, 2 × 10^4^ U87MG cells or 2 × 10^5^ SVEC4-10EE2 MECs were seeded in a six-well plate and allowed to adhere. Twenty-four hours later, these cells were treated with H1/Vastatin or H1/EGFP (N/P ratio = 20) for 6 h at a dosage of 10 μg DNA per well and then incubated in DMEM with 10% FBS. Cell viability was assessed 2, 4, or 7 days later by trypan blue exclusion and viable cells were counted manually [21]. In the co-culture system, 2 × 10^5^ MECs were seeded in a six-well plate while 2 × 10^5^ U87MG cells were seeded onto the inner surface of the PET membrane located at the base of the Falcon^TM^ culture insert (BD Biosciences). The insert was then placed into the six-well plate where the MECs were seeded. H1/Vastatin or H1/EGFP treatment was added to the inner surface of the insert for 6 h. Proliferation assays were carried out by counting the viable MECs at the same aforementioned time points.

To evaluate the inhibitory effects of secreted Vastatin on MEC proliferation, conditioned media were used. In brief, 2 × 10^6^ U87MG or SVEC4-10EE2 cells were seeded in 100 mm culture dishes, treated with H1-Vastatin or H1-EGFP at a dose of 10 μg DNA per dish for 8 h, then incubated in DMEM with 10% FBS for 96 h. The conditioned media were collected and centrifuged at 600 g, 4 °C for 10 min. SVEC4-10EE2 MECs were seeded in a 96-well plate at a density of 5000 cells per well. After cell attachment, the media were changed to serial dilutions of conditioned media with 10% FBS. Seven days later, 3-(4,5-dimethyl-2-thiazolyl)-2,5-diphenyl-2H-tetrazolium bromide (MTT) was added to the media and incubated for 2 h. The media were then changed to dimethyl sulfoxide (DMSO) and assessed by colorimetric analysis at 570 nm.

### In vitro temozolomide resistance testing

For proliferation inhibition, 2000 U87MG or U87-ATR cells were seeded into each well of a 96-well plate and treated with increasing concentrations of TMZ. MTT assays were used to examine cell viability 4 days later. For clonogenic survival assays, U87MG or U87-ATR cells were seeded into a six-well plate at a density of 500 cells per well. The media were then changed to DMEM containing 10% FBS and 100 μM TMZ for incubation. On day 14 the number of colonies containing more than 50 cells were counted.

### Statistical analysis

Mice survival was analysed with PASW Statistics Version 18 (SPSS Inc., Chicago, Illinois). Comparisons in proliferation tests and MVD analysis were conducted by one-way analysis of variance or two-tailed Student’s t test. Comparisons of animal survivals were performed using Log-rank test. *P* < 0.05 was considered statistically significant.

## Results

### H1-Vastatin transfected GB cells and inhibited MECs proliferation through paracrine suppression

**Fig. 1:**
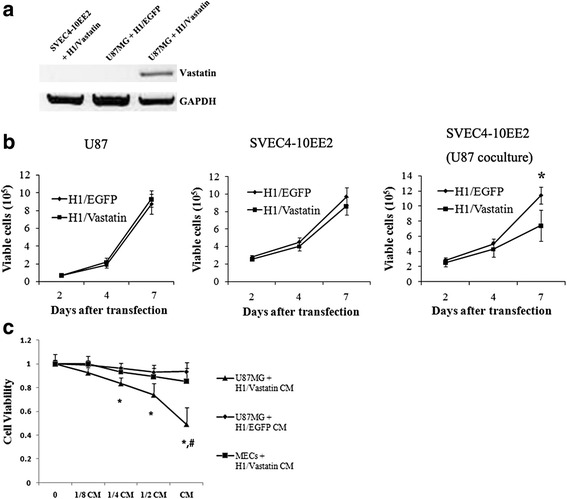
H1-Vastatin transfectd tumour cells specifically and suppressed MECs proliferation through paracrine inhibition. **a** H1 mediated gene transfections targeted only the tumour cells. Enhanced transcription level of Vastatin was only detected in the H1-Vastatin treated U87MG cells. **b** Proliferation curves of cells treated by H1-Vastatin or H1-EGFP. H1-Vastatin did not affected the proliferation of U87MG cells or MECs in separate culture condition. In the U87MG and MECs co-culure system, H1-Vastatin significantly suppressed the MECs growth on day 7 post treatment (*P* < 0.05). **c** Inhibition curves showing effects of different conditioned media (CM) on MECs proliferation. Conditioned medium from H1-Vastatin treated U87MG cells significantly decreased the cell viability of MECs in a dosage dependant way (* *P* < 0.05 against CM from H1-EGFP treated U87MG cells; # *P* < 0.05 against CM from H1-Vastatin treated MECs), suggesting Vastatin secreted by tumour cells inhibited neovascularization in paracrine manner

The H1 gene vector was designed specifically to target tumour cells [18]. We proposed that H1 mediated gene transfection could restrict the expression and secretion of therapeutic agents in tumour areas and prevent systemic side effects. To prove this idea, GB U87MG cells and mouse MECs SVEC4-10EE2 were treated with either H1-Vastatin or H1-EGFP in culture. Only U87MG cells treated by H1-Vastain showed enhanced Vastatin mRNA levels (Fig. 1a), which was consistent with previous report that H1-DNA nanoparticles transfected cancer cells specifically. We further conducted a series of proliferation tests on days 2, 4 and 7 after the cells received H1-Vastatin treatment. As anticipated, H1-Vastatin showed no significant influence on cell viability of either U87MG or MECs (Fig. 1b, *left and middle*). However in a U87MG and MECs trans-well co-culture system, the proliferation of MECs was significantly inhibited on day 7 post-transfection (Fig. 1b, *right*; *P* < 0.05). This has demonstrated that the expression and secretion of Vastatin from H1-Vastatin transfected U87MG cells was necessary for inducing proliferation inhibition in MECs. Then we collected conditioned media (CM) from different culture groups on day 4 post-treatment. MECs SVEC4-10EE2 were seeded into a 96-well plate and incubated in serial dilutions of these CM. Proliferation test was performed 1 week later. Cell viabilities from different culture conditions were normalized to the NO CM culture group to generate an inhibition curve (Fig. 1c). The results showed that CM collected form H1-Vastatin treated U87MG reduced MECs proliferation in a dose-dependent way, while CM from H1-Vastatin treated MECs or H1-EGFP treated U87MG had no such effect. This further supported our anticipation that H1-Vastatin could induce Vastatin secretion from tumour cells and suppress MECs proliferation by paracrine inhibition.

### Administration of H1-Vastatin prolonged survival in GB-bearing mice

**Fig. 2:**
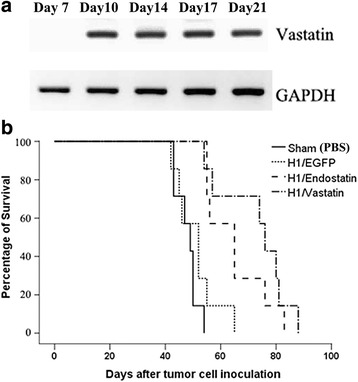
Administration of H1-Vastatin increased intracranial Vastatin expression and significantly prolonged survival of GB bearing mice. **a** Bands of Vastatin exclusive PCR products in agarose gel electrophoresis. H1-Vastatin significantly enhanced the mRNA level of Vastatin in the right hemispheres of treated mice, which lasted over 2 weeks. **b** Survival curves of GB bearing mice (*n* = 7). H1-Vastatin and H1-Endostatin treatment significantly prolonged the median survival time of GB bearing mice to 75 and 64 days respectively, from 48 and 51 days for the PBS and H1-EGFP treated groups (*P* < 0.05). There was no significant difference in survival time between H1-Vastatin and H1-Endostatin treated groups

The therapeutic benefit of H1-Vastatin was studied on GB-bearing mice and compared with PBS, EGFP and Endostatin. An orthotopic GB model was established by intracranial inoculation of U87MG cells into the nude mice. Intracerebral injections of H1-Vastatin, H1-Endostatin, H1-EGFP or PBS were performed on day 7 and day 14 after cells inoculation. Sustained expression enhancement of intracranial Vastatin level was observed after H1-Vastatin treatment (Fig. 2a). H1-Vastatin successfully prolonged animal survival from a median of 48 days (PBS treated group) to 75 days (*P* < 0.01, *n* = 7 for each group; Fig. 2b). The animal survival was also significantly extended in the Endostatin treated group (median survival of 64 days; *P* < 0.01 against the PBS treated group). H1-EGFP caused no significant difference on animal survival (median survival of 51 days), suggesting that the vector per se did not interfere with the test. However, no significant difference in animal survival was detected between the Vastatin group and the Endostatin group. These results imply that Vastatin has a potent anti-tumour activity in this GB model, and is comparable to the well studied endogenous antiangiogenic agent Endostatin.

### Administration of H1-Vastatin decreased microvessel density (MVD) in GB-bearing mice

**Fig. 3:**
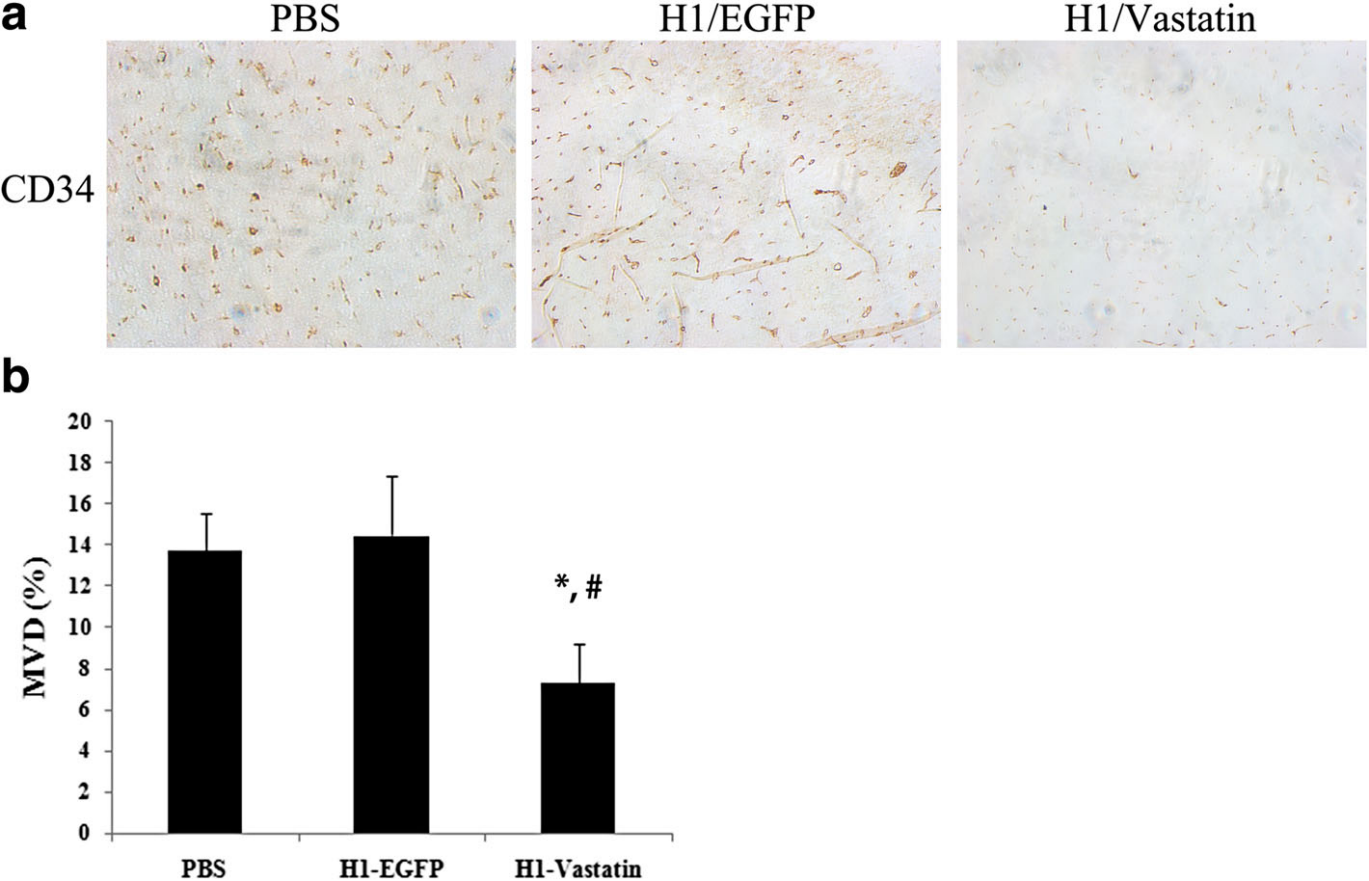
H1-Vastatin caused angiogenesis inhibition in vivo and decreased microvessel density. **a** Immunostaining of CD34, a vessel endothelia marker, on brain tumour sections. The CD34+ cell numbers were fewer in the H1-Vastatin treated group than the PBS and H1-EGFP treated groups. **b** Histogram showing the microvessel density (MVD) in tumours of different treatment groups. Percentage of microvessel endothelial cells in the H1-Vastatin treated group was significant lower than in the PBS and H1-EGFP treated groups. (*n* = 3; * *P* < 0.05 against PBS treated group, # *P* < 0.05 against H1-EGFP treated group)

For histological assessment, mice bearing GB xenografts were treated with PBS, H1-EGFP or H1-Vastatin respectively (*n* = 3 for each group). The animals were sacrificed at day 42 post tumour cell inoculation. The brain tissues were then fixed in formalin, embedded into paraffin blocks and processed for slicing and staining. Angiogenesis was detected by immunohistochemical staining against cells expressing CD34, a protein marker for blood vessel endothelial cells. Results showed that H1-Vastatin significantly reduced CD34+ cells in brain tumours (Fig. 3a). Microvessel density in the H1-Vastatin treated group (7.3 ± 1.9) was significantly lower than those in the PBS (13.7 ± 1.8, *P* < 0.05) and H1-EGFP (14.5 ± 2.9, *P* < 0.05) treated groups (Fig. 3b). These results indicated that Vastatin induced angiogenesis inhibition and eliminated tumor microvessels in the orthotopic GB model, which could be the underlying mechanism of its survival benefits.

### H1-Vastatin synergized with TMZ in chemoresistant GB model

**Fig. 4:**
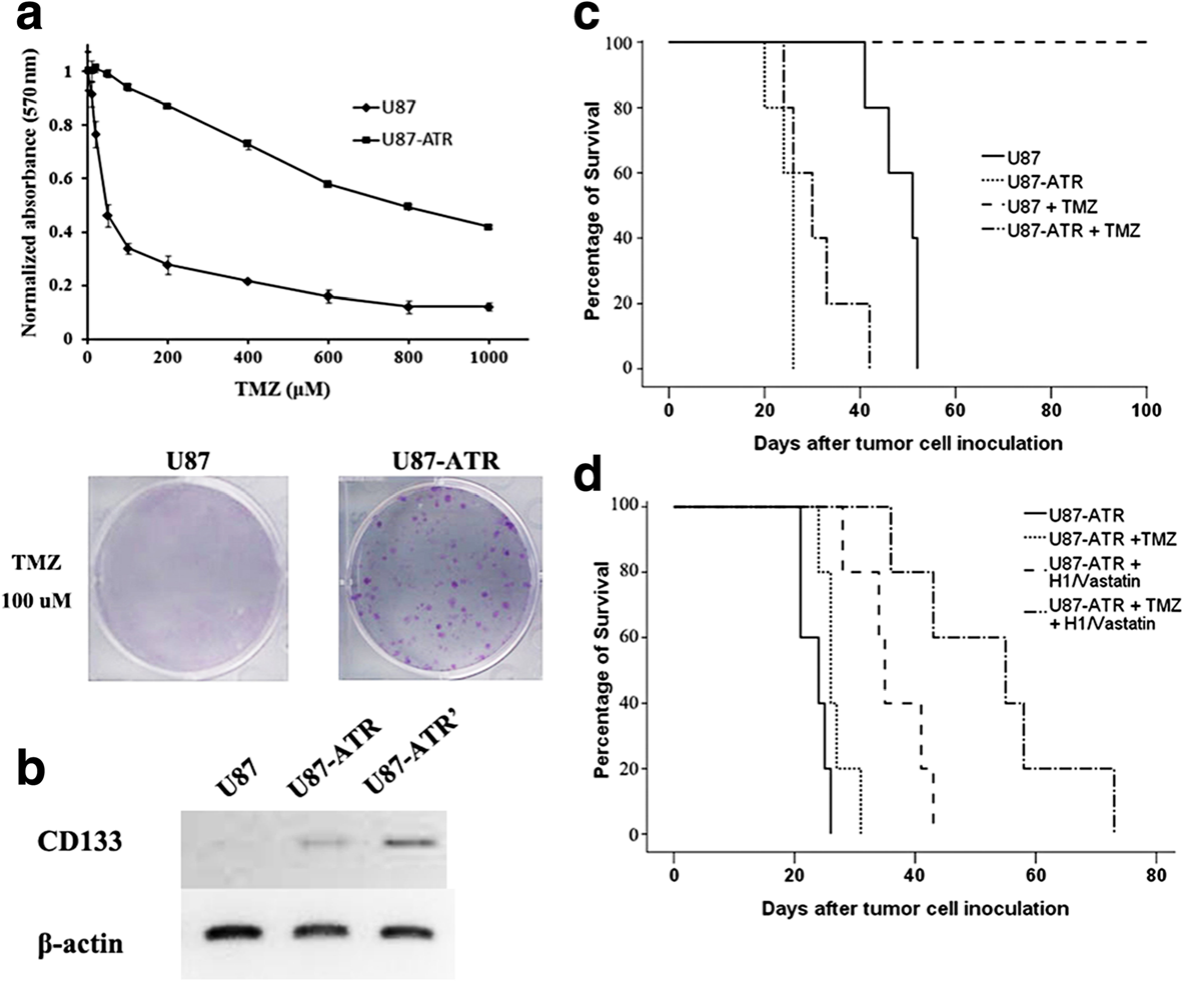
Vastatin synergized with temozolomide in GB chemoresistant model. **a** TMZ resistance of U87-ATR cells. U87-ATR had a much higher half inhibited dosage of TMZ (>800 μM) than U87MG (<50 μM) in the proliferation test (*upper*). More U87-ATR than U87MG cells survived the treatment of 100 μM TMZ and formed cell colonies (*lower*). **b** U87-ATR cells showed enhanced cancer stem cell property by expression of CSC marker CD133. U87-ATR’ were cells amplified from a single cell clone which was picked out from TMZ treated U87-ATR. **c** Survival curves showing that GB model established using U87-ATR had a much shorter survival time (25 days) than using U87MG cells (50 days, *P* < 0.05), and did not respond to TMZ treatment (*n* = 5). **d** Survival curves of TMZ resistance GB animals treated by H1-Vastatin and/or TMZ (*n* = 5). H1-Vastatin significantly prolonged the median survival of animals bearing U87-ATR xenografts to 34 days (*P* < 0.05). The combination of TMZ and H1-Vastatin showed even better therapeutic effects, with median survival extended to 54 days (*P* < 0.01 against H1-EGFP treated group; *P* < 0.05 against H1-Vastatin single treatment group). This result suggested Vastatin synergized with TMZ and restored the sensitivity of chemoresistant mice to TMZ treatments

TMZ is an alkylating agent which damages tumor cell DNA and triggers cell death. It has been demonstrated to confer moderate survival benefits for GB patients. To test whether H1-Vastatin could facilitate current management of GB, we performed a combined treatment of Vastatin and TMZ on GB orthotopic model. We also established a TMZ resistant GB model, since intrinsic and acquired chemoresistance are the main clinical challenges encountered in TMZ therapy. To induce a stable TMZ resistant cell line, U87MG cells were exposed to TMZ containing medium for a long term incubation. The generated cells, named U87-ATR, were confirmed to be TMZ resistant in both proliferation and survival assays (Fig. 4a). Nude mice intracranially inoculated with U87-ATR cells had significantly shorter survival (median survival of 25 days, *n* = 5) than those with U87MG cells (median survival of 50 days; *P* < 0.05). TMZ was found extremely effective in treating U87MG bearing mice, with all the animals in this group survived the total duration of 100 days. In contrast, mice with U87-ATR xenografts showed no significant response to TMZ treatment (median survival of 29 days; Fig. 4c). Our results further showed that H1-Vastatin was effective in the treatment of this TMZ resistant model, and significantly extended the median survival from 23 days of the H1-EGFP treated group to 34 days (Fig. 4d; *n* = 5, *P* < 0.05). More interestingly, a synergistic effect was noted between H1-Vastatin and TMZ, which further prolonged the median survival to 54 days (*P* < 0.01 against H1-EGFP treated group; *P* < 0.05 against H1-Vastatin single treatment group).

## Discussion

Angiogenesis is the physiological process by which new blood vessels develop from pre-existing vessels. In normal tissues, it is precisely regulated by a series of angiogenic stimulators and inhibitors. In the state of tumour growth, the balance between the stimulators and inhibitors is tipped, towards an “angiogenic switch” [22]. VEGF is one of these stimulators and plays a predominant role in regulating tumour angiogenesis. A humanized monoclonal antibody against VEGF, bevacizumab, has been shown to exhibit treatment response resulting in a longer progression-free survival in GB patients [23, 24]. However, angiogenic inhibitors like bevacizumab which target a single pathway often encounter rapid onset resistance through alternative pathways [25]. Studies that aimed to overcome this resistance have suggested the utilization of a combination of single-pathway targeted antiangiogenic agents [26]. Another alternative is to use broad-spectrum antiangiogenic agents. Endostatin, for example, is a 20-kDA C-terminal cleavage fragment of collagen type XVIII and possesses the broadest anti-cancer spectrum. It targets angiogenesis regulatory genes that comprise of more than 12% of the human genome [27]. Approved by the State Food and Drug Administration of the People’s Republic of China, Endostatin is currently a treatment option for non-small-cell lung cancer. Several reports also suggest that Endostatin might be effective in inhibiting tumour growth in malignant glioma in animal models [28–30].

Endostatin represents a group of endogenous angiogenic inhibitors that are fragments of larger extracellular matrix (ECM) molecules. During angiogenesis, the breakdown of the ECM is a prerequisite for the initiation of sprouting. Endogenous antiangiogenic components are released during this process and act as focal natural feedback [15]. Among them are the NC1 domains cleaved from collagen molecules. Endostatin is the NC1 domain of collagen XVIII. Others include Arresten, Canstatin and Tumstatin from collagen IV, Restin from collagen XVα1, and Vastatin from collagen VIII [13, 31–34]. They form a family collectively referred to as collagen-derived antiangiogenic factors (CDAFs). In cancer studies CDAFs have been reported to be effective in suppressing tumour progression, both in vitro and in vivo [35–37]. Furthermore, these endogenous inhibitors, having been demonstrated to be safe, acting on multiple proangiogenic pathways, are therefore attractive therapeutic candidates [38, 39].

Vastatin is a CDAF from type VIII collagen. Type VIII collagen is present in the ECM of sclera, skin and the renal glomerulus participating in their vascularization [40]. In contrast, Vastatin, contributes to the suppression of ocular neovascularization [15]. The potential of Vastatin in tumour treatment is not fully explored, even though type VIII collagen is highly expressed in selected solid tumours. As far as we know, we are the first to introduce Vastatin into preclinical malignant tumour studies. In our previous report, Vastatin is absent in human HCC, and rAAV-Vastatin infection effectively inhibites proliferation, migration and microvessel formation activities in MECs [14]. In this study we further demonstrate that Vastatin can inhibit angiogenesis and may be of therapeutic benefit in GB. Mechanism studies from our previous HCC research showed that Vastatin inhibited cellular metabolism, Notch and AP-1 signaling pathways [14]. Considering this result was from an in vitro study using MECs not specifically originated from HCC, we believed it could also be used for explaining the Vastatin induced antiangiogenesis in the GB model. The Notch signaling pathway in tumour angiogenesis is well-characterized. In general, delta-like ligand 4 (Dll4) interacts with Notch receptors and reduces VEGF signal transduction on stalk cells during sprouting, which contributes to the structural and functional integrity of newly formed vessels [41]. Inhibition of Dll4 and Notch signaling leads to functionally compromised vessels and suppresses tumour growth [42]. This may help to explain why Vastatin aggravated necrosis in our previous HCC study [14]. Changes in the degree of necrosis was not so obvious in current GB study, probably because the nature of the tumour inherently exhibits an abundance of necrosis as a hallmark feature. In GB, the Notch ligands provided by endothelial cells were also shown to be important for maintaining cancer stem-like cells (CSLCs) [43]. Inhibition of Notch signaling may cause growth inhibition of GSCs [44], which we believed was a possible mechanism underlying Vastatin’s anti-glioma effect and distinguished Vastatin from traditional antiangiogenic agents. Unlike Notch signaling, the down-regulation of AP-1 and cell metabolism pathways seems to have a more direct influence on reducing MEC viability. AP-1 is a transcription factor that regulates a wide range of cellular processes, including cell growth, differentiation and apoptosis. In GB, it mediates anoxia induced up-regulation of interleukin-8 (IL-8), a tumourigenic and proangiogenic chemokine [45]. In addition, AP-1 is involved in epidermal growth factor receptor (EGFR) mediated TMZ resistance [46]. Although we did not investigate the relationship between endothelium metabolism and antiangiogenic therapies, it was generally accepted that insufficient nutrients metabolism would lead to cell cycle arrest and apoptosis [47]. This is substantiated by evidence showing that enhanced glucose and glutamine metabolism in proliferating endothelial cells promotes tumour angiogenesis [48]. Altogether these findings depict a multi-targeted antiangiogenic pattern for Vastatin and considerably promotes its potential as an effective therapy for GB.

Safety is a primary concern in the treatment of brain tumours. Vastatin has been proven to be generally safe for systemic administration in previous HCC study [14]. However in this report, we highlighted the feasibility of recruiting H1 for local administration of antiangiogenic therapeutics. H1 induces endocytosis by binding to folate receptors that are highly expressed on certain tumour cell surfaces but not MECs [18]. Both the co-culture and conditioned medium test results imply that H1-Vastatin induced inhibition of MECs proliferation can be achieved by Vastatin secreted from adjacent GB tumour cells. In other words, H1-Vastatin selectively infects GB cells, restricting its antiangiogenic effects to the vicinity of the tumor thereby reducing the possibility of systemic adverse effects. Our observations that no deleterious effects were detected during the subsequent animal study is consistent with this hypothesis. This type of paracrine inhibition is also compatible with the “angiogenic switch” theory and restores the balance between angiogenic stimulators and inhibitors in the perivascular tumor microenvironment.

Whether antiangiogenic treatments could promote or attenuate chemotherapies is controversial, since changes in vascular integrity and permeability might complicate the passing of medications across the blood brain barrier. Clinical studies have combined bevacizumab with different cytotoxic chemotherapeutic agents in the treatment of either primary or recurrent GB. The results, unfortunately, were negative [12, 49, 50]. Nevertheless, the present study showed H1-Vastatin had a significant synergistic effect with TMZ in a chemoresistant GB murine model. This might be explained by the difference in anti-angiogenic mechanisms of bevacizumab and Vastatin, especially with regards to the Notch signaling regulation. Notch ligands expressed by endothelial cells are crucial for maintaining self-renewal of cancer stem cells [43]. Notch signaling pathway inhibition coupled with TMZ has been proven to exert an anti-glioma stem cell effect [51]. Moreover, the negative Notch-1 expression state was associated with longer patient survival [52]. During the development of our mouse model, we introduced a group of cells with acquired TMZ resistance from original U87MG cells. These U87-ATR cells exhibited significant stem cell properties as evidenced by the high expression of cancer stem cell marker CD133 (Fig. 4b). The synergistic effect between Vastatin and TMZ in U87-ATR bearing mice might possibly be mediated by the suppression of Notch signaling in MECs, which subsequently lead to the eradication of perivascular niches for U87-ATR and other chemoresistant cancer stem like cells. However, it is one of our limitations that we did not show a direct inhibition effect of Vastatin treated MECs on U87-ATR cells, due to the lack of efficient cell-cell interaction model as well as the complications caused by the paracrine angiogenesis inhibition strategy. Studies to further investigate the synergistic effect between Vastatin and TMZ are ongoing, which we believe will help to discover the underlying mechanisms not just limited to a single pathway.

## Conclusion

We report for the first time that Vastatin can induce antiangiogenesis and prolong survival in mice bearing GB orthotopic xenografts. We also confirm that H1-Vastatin offers a safe and efficient targeting method for GB antiangiogenic therapeutic tests. More importantly, a synergistic treatment effect is observed when Vastatin is coupled with TMZ therapy, which leads to the resensitization of initial chemoresistant GB model to TMZ treatment. At present, there is no effective treatment for patients with recurrent GB. Our results regarding the anti-tumor effects of Vastatin bear potential clinical therapeutic significance. The limitation of this study was that only used one animal model with one GB cell line were employed. Future studies should confirm these findings in models with more cell lines and different animals. Studies are also needed to further elucidate the pharmacological properties of Vastatin and its toxicological profile. In addition, combination effect of Vastatin and radiotherapy should be tested, since radiotherapy is a first-line treatment to GB patient and radioresistance is related to CSCs as well.

## Abbreviations

AP-1: Activator protein-1
ATR: Acquired temozolomide resistance
CDAF: Collagen-derived antiangiogenic factor
CM: Conditioned medium
CSC: Cancer stem cell
ECM: Extracellular matrix
EGFP: Enhanced Green Fluorescent Protein
GB: Glioblastoma
HCC: Hepatocellular carcinoma
MEC: Microvessel endothelial cell
MVD: Microvessel density
PBS: Phosphate buffered saline
PEI: Polyethylenimine
rAAV: Recombinant adeno-associated virus
TMZ: Temozolomide
VEGF: Vascular endothelial growth factor
